# Proportions and Seasonal Patterns of Respiratory Viruses via Rapid Tests in Adults at a Greek Hospital (Oct. 2023–Mar. 2024)

**DOI:** 10.3390/jpm14080824

**Published:** 2024-08-03

**Authors:** Eleni Rousogianni, Garyfallia Perlepe, Stylianos Boutlas, Georgia G. Rapti, Evdoxia Gouta, Eleni Mpaltopoulou, Giorgos Mpaltopoulos, Erasmia Rouka, Dimitrios Papagiannis, Konstantinos I. Gourgoulianis

**Affiliations:** 1 Department of Respiratory Medicine, Faculty of Medicine, School of Health Sciences, University of Thessaly, 41500 Larissa, Greecebaltopoulosgiorgos@gmail.com (G.M.); kgourg@uth.gr (K.I.G.); 2 Emergency Department, University Hospital of Larissa, 41334 Larissa, Greece; 3 Department of Nursing, School of Health Sciences, University of Thessaly, 41110 Larissa, Greece; 4 Public Health & Vaccines Laboratory, Department of Nursing, School of Health Sciences, University of Thessaly, 41110 Larissa, Greece; dpapajon@gmail.com

**Keywords:** influenza A/B, SARS-CoV-2, RSV, adenovirus, rapid test, epidemiology, seasonality, respiratory infections

## Abstract

Background: Respiratory infections pose a major public health threat. The predominant viruses causing viral respiratory infections are influenza A and B (Flu-A, Flu-B), coronaviruses, respiratory syncytial virus (RSV), and adenovirus. This study aims to investigate the proportion of these cases via rapid antigen tests and assess seasonal patterns. Methods: Clinical samples were collected from symptomatic adults presenting to the Emergency and Respiratory Medicine Departments of the University Hospital of Larissa (UHL), Greece from 16 October 2023 to 31 March 2024. Nasal specimens were antigen-tested for Flu-A/B, SARS-CoV-2, RSV, and adenovirus. Results: The total sample of specimens collected was 1434, of which 739 (51.5%) were female and 695 were male (48.5%). The mean age of participants was 57 ± 5.5 years. Among the positive results, we recorded a proportion of 40.18% and 11.40% for influenza A and B, respectively, followed by 35.79% for SARS-CoV-2, 10.70% for RSV, and 1.93% for adenovirus. Conclusions: In Greece, surveillance systems in infection control are underutilized. Rapid tests via multiple antigens can quickly identify viral infections, making them a valuable tool with financial benefits for health systems. Early detection of respiratory infections helps allocate resources efficiently, ensures adequate staff and facilities are available, and improves patient care through refined clinical management.

## 1. Introduction

In recent times, the outbreak of various infectious diseases has deeply affected millions of lives, straining medical facilities and burdening economists, scientists, and politicians [1]. Various microorganisms cause acute respiratory tract infections (ARTIs); however, the most contagious and medically urgent infections are often associated with respiratory viruses (such as RSV, Influenza A or B, and coronaviruses). The recent COVID-19 pandemic and the memory of the Spanish flu (the 1918 influenza pandemic) are stark reminders of the dangers caused by highly contagious viruses. Over the years, an important number of deadly outbreaks of severe influenza and coronaviruses have arisen [1]. In this context, monitoring respiratory infections poses a challenge for public health authorities.

Seasonal influenza is a highly contagious annual respiratory virus that circulates worldwide, significantly increasing morbidity and mortality. There are four types of influenza viruses: A, B, C, and D. Types A and B cause seasonal epidemics, with type A known for causing pandemics. Type C typically results in mild infections and is not a major public health concern, while type D does not infect humans [2]. Influenza viruses undergo minor changes (“antigenic drift”), leading to seasonal outbreaks that typically last 8–10 weeks from late autumn to early spring [3]. Between 2010 and 2023, the CDC estimates that influenza caused 9.3–41 million illnesses, 100,000–710,000 hospitalizations, and 4900–51,000 deaths annually [4]. Occasionally, influenza viruses undergo major changes (“antigenic shift”), which can lead to pandemics. These shifts often originate from nonhuman sources and can trigger pandemics due to a lack of immunity. Rapid detection and reporting are crucial for prompt public health responses. Most individuals recover from symptoms such as fever, dry cough, headache, muscle pain, fatigue, sore throat, and runny nose within a week without medical attention. However, influenza infection can lead to hospitalization or death in high-risk groups (children < 3 years, individuals > 60 years, pregnant women, and people with chronical medical conditions) [2].

The COVID-19 pandemic caused by SARS-CoV-2 continues to cause significant morbidity and mortality all over the world and significantly burdens healthcare systems, leading to substantial economic losses [5]. In late 2019, the Wuhan Municipal Health Commission, China reported a cluster of cases of pneumonia in Wuhan, Hubei Province. The cause of the pneumonia was eventually identified as a novel coronavirus, which rapidly spread across the globe, leading to its official declaration as a pandemic on 11 March 2020 [6]. As of 5 May 2024, the World Health Organization (WHO) reported that there have been 279,350,756 cases of COVID-19 in Europe and 775,431,269 cases worldwide. Additionally, approximately 900,000 deaths have occurred globally due to the virus. SARS-CoV-2 typically spreads via respiratory droplets, and common symptoms include fever, cough, dyspnea, myalgia, or fatigue. In humans, infections can range from asymptomatic to severe pneumonia, especially in the elderly, the immunocompromised, and people with respiratory diseases, potentially requiring hospitalization and sometimes leading to death [7]. Children generally experience milder disease compared to adults [8].

While RSV is identified as an important respiratory pathogen in infants causing lower respiratory infections, it is often overlooked in older adults, with its disease burden among those aged ≥ 60 years in high-income countries being higher than previously thought [9]. Generally, in countries with temperate climates, RSV is particularly prevalent during the winter season with one of the two genotypes (A and B) predominating in a single season, alternating annually [9]. Among adults, RSV manifests with a broad spectrum of clinical symptoms, including upper respiratory tract infections, severe lower respiratory tract infections, and exacerbations of underlying diseases [9]. RSV constitutes a significant risk factor of disease and mortality in children, older individuals, and immunocompromised patients, while its impact on adults admitted to the hospital is highlighted by the expanded use of multiplex molecular assays [10].

Human adenoviruses (HAdVs) are DNA viruses that account for a significant proportion of respiratory tract infections (RTIs) throughout the year, contributing to up to 10% of pediatric cases and up to 7% of adult cases [11,12]. Common symptoms of HAdV RTI include fever, pharyngitis, tonsillitis, cough, and sore throat, sometimes leading to pneumonia [12]. With approximately 60 known genotypes, HAdVs present a wide clinical spectrum, ranging from mild respiratory illness, gastroenteritis, and conjunctivitis to more severe conditions or even death, especially in vulnerable populations [12,13]. In immunocompromised individuals, HAdV infections can lead to dissemination and severe respiratory failure in 10–30% of cases, with fatality rates for severe HAdV pneumonia potentially exceeding 50% [12]. However, HAdVs are often underreported and less studied compared to other respiratory viruses [14].

Viral infection symptoms often mimic those of bacterial infections, complicating efforts by public health authorities to distinguish between them. The main viruses causing acute respiratory infections are Flu-A, Flu-B, RSV, and coronaviruses. Diagnosing these infections promptly is crucial for better clinical outcomes but can be challenging. Rapid antigen tests for multiple pathogens offer a reliable, fast, and cost-effective method for detecting viral infections. Early and accurate diagnosis can help reduce the misuse of antibiotics and enable the use of antiviral treatments [15].

This cross-sectional study was designed to investigate the epidemiological prevalence of specific respiratory viruses among symptomatic adults during the winter-spring period of 2023–2024 and to evaluate the seasonal patterns of these viruses.

## 2. Materials and Methods

In this cross-sectional study, nasopharyngeal specimens were utilized as part of a surveillance program conducted in the Emergency Department (ED) and Respiratory Medicine Department of the University Hospital of Larissa (UHL). The study period encompassed all on-call days between 16 October 2023 and 31 March 2024. According to the duty schedule, UHL receives patients in 24 h turns (i.e., every second day), and therefore, data on the variables of interest were available every two days.

Nasopharyngeal samples were systematically collected from adults presenting symptoms consistent with respiratory infection, including fever, cough, malaise, sore throat, runny nose, headache, shortness of breath, and chest pain within the preceding 10 days upon hospital presentation. The participants were informed about the study’s objectives by the attending physicians, and demographic data, including age and gender, were reported. The eligibility criteria to participate in the present study were the presence of respiratory infection symptoms within the preceding 10 days, adulthood, and provision of consent to participate. The study was conducted according to the guidelines of the Declaration of Helsinki and approved by the Institutional Review Board (or Ethics Committee) of the University of Thessaly (approved on 16 October 2023). Written informed consent was obtained from the patients. The statistical analysis used Excel 2019 (Microsoft, Redmond, Washington, DC, USA) and IBM SPSS (version 26, SPSS, Chicago, IL, USA).

The collected samples underwent antigen testing for the detection of four different pathogens. To streamline the process, a simplified specimen collection method was introduced, requiring only one swab instead of four separate samples for the detection of all four pathogens. This approach minimizes discomfort, offers a more cost-effective solution, reduces the demand for personnel, and alleviates unnecessary stress for all involved parties.

### Principle of Assay

Antibodies specific to viral proteins are applied to the test line region of the nitrocellulose membrane. During testing, viral antigens in the specimen interact with the antibodies conjugated to gold nanoparticles. As the sample flows through the test membrane, it moves upward and reacts with the antibodies immobilized on the membrane, producing a colored line in the test region. The presence of this colored line signifies a positive result. A colored line in the control region always appears if the test has been correctly performed, serving as a procedural control The test results in this study were interpreted after 15 min.

The four rapid diagnostic tests (RDTs) used in this study were manufactured by PROGNOSIS BIOTECH S.A (Larissa, Greece) and met the requirements of EN ISO 13485:2016 [16].

These tests were used in a previous cross-sectional study published recently and demonstrate high sensitivity and specificity [5]. That study aimed to record the proportion of RSV, SARS-CoV-2, influenza A/B, and adenovirus cases using rapid antigen tests and validate the results with RT-PCR assays of upper respiratory specimens among 784 children in the community during Winter 2023 in central Greece.

## 3. Results

The sample of specimens collected included 1434 adults, consisting of 695 men and 739 women. The mean age of the participants was 57 ± 5.5 years. Among 570 (39.74%) positive results, we recorded a proportion of 40.18% (N = 229) for Flu-A, 11.40% (N = 65) for Flu-B, 35.79% (N = 204) for SARS-CoV-2, 10.70% (N = 61) for RSV, and 1.93% (N = 11) for adenovirus. 864 adults (60.25%) yielded negative rapid test results. These patients either tested positive for non-targeted pathogens or had no identifiable pathogens.

**Figure 1:**
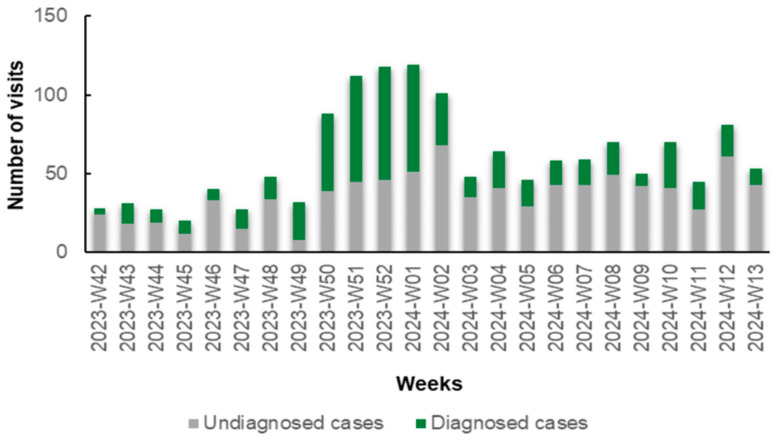
Number of diagnosed (green columns) and undiagnosed (gray columns) cases of respiratory viruses via rapid antigen tests in patients with symptoms of respiratory infection presenting to the Emergency Department (ED) and Respiratory Medicine Department of the University Hospital of Larissa, Greece, between 16 October 2023 (W42), and 31 March 2024 (W13), by epidemiologic week.

Notably, during the initial weeks (W42–W48, 2023) and the concluding weeks (W02–W13, 2024) of the study, the number of patients diagnosed with specific antigens through rapid tests was lower compared to those who remained undiagnosed. Conversely, in the intervening period (W49, 2023 to W01, 2024), this trend reversed, with a predominance of diagnosed cases over undiagnosed ones (Figure 1). This reversal is primarily attributed to the prevalence of influenza A during this period. In detail, during W42–W48 and W02–W13, 291 patients received a diagnosis (30.16%) and 674 remained undiagnosed (69.84%) via rapid antigen testing. From W49 to W01, 279 (59.49%) received a diagnosis and 190 (40.51%) remained undiagnosed via rapid antigen testing.

**Figure 2:**
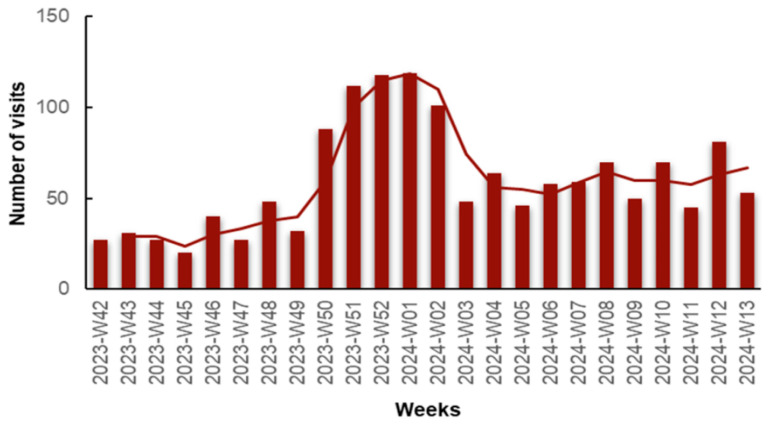
Number of visits for respiratory infection symptoms to the Emergency Department (ED) and Respiratory Medicine Department of the University General Hospital of Larissa, Greece, from 16 October 2023 (Week 42) to 31 March 2024 (Week 13), by epidemiologic week with a 2-period moving average line.

We observed an increase in visits in early December, peaking at weeks 52, 2023 and 01, 2024 (late December, early January). This significant rise can be attributed to the increase in influenza A-positive and COVID-19-positive cases, as we will elaborate on later. A second spike in visits was observed during weeks 08 to 13, 2024, possibly attributed to the Flu-B wave, as we will discuss further (Figure 2).

**Figure 3:**
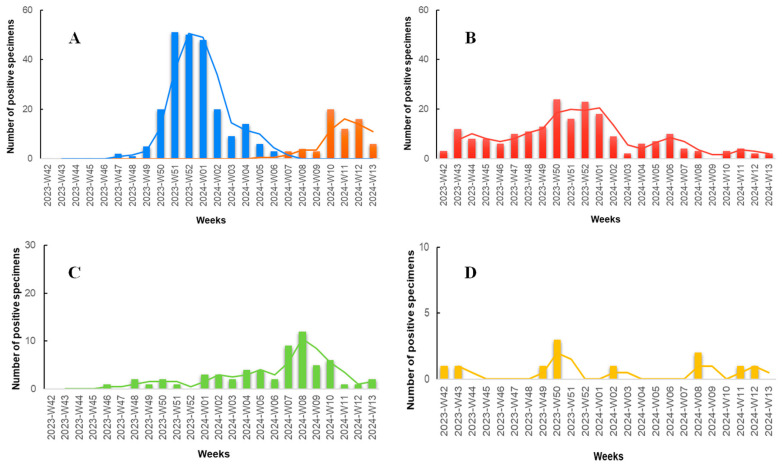
Number of positive rapid antigen test results positive for various respiratory pathogens among patients presenting with symptoms of respiratory infection to the Emergency Department (ED) and Respiratory Medicine Department of the University Hospital of Larissa, Greece between 16 October 2023 (W42), and 31 March 2024 (W13). The data are segmented by epidemiologic week and shown in four bar charts, each with a 2-period moving average line and individual scaling to best represent the data. (**A**) Influenza A (blue columns) and influenza B (orange columns). (**B**) SARS-CoV-2 (red columns). (**C**) RSV (green columns). (**D**) Adenovirus (yellow columns).

According to the results of our study (Figure 3), during the initial weeks, we observed a minimal number of specimens positive for the targeted pathogens, with the majority being positive for SARS-CoV-2. In late November (Week 47, 2023), the first specimen positive for influenza A was documented, marking the onset of an abrupt increase in influenza A specimens, peaking in late December (Weeks 51–52, 2023). Concurrently, during the Flu-A wave, the number of SARS-CoV-2 positive specimens also increased, with a lower peak compared to influenza A occurring in mid to late December (Weeks 50–52, 2023). Throughout the study period, COVID-19 seemed to exhibit consistently low but measurable positive specimens. RSV was also detected at low levels throughout the study period but demonstrated an increase in mid-February to early March (Weeks 07–10, 2024). The Flu-B wave occurred later, with the first positive specimen recorded in late January (Week 05, 2024), peaking in March (Weeks 10–12, 2024), and exhibiting detectable rates even in the last week of the study period. Adenovirus documented the lowest rates compared to the other viruses, with no noticeable seasonal pattern observed.

## 4. Discussion

Many factors can cause the seasonality of respiratory viruses, such as changes in climate that affect how viruses spread, changes in host immunity (e.g., decreased immunity from past infections or vaccines), and changes in human behavior during winter [17]. Understanding the seasonality of these pathogens is crucial for implementing targeted interventions to reduce the strain on our healthcare system when it is most needed [18].

Influenza is a common respiratory infectious disease that circulates globally, affecting millions of people annually. The timing of the annual “flu season” also varies from season to season. Flu viruses are most active during the fall and winter, typically rising in October and peaking between December and February, although activity can last until May [19].

Before the COVID-19 pandemic, influenza activity typically peaked later, such as during the 2017–2018 and 2018–2019 flu seasons, with peaks occurring around weeks 3 to 5 in 2018 and weeks 8 to 10 in 2019, respectively [20]. The pandemic led to a decline in influenza activity [21]. However, during the 2022–2023 season, an early onset and peak were observed, with activity levels similar to pre-pandemic times [20,22]. For the 2023–2024 season, data from the CDC, ERVISS, and our study show alignment and some differences. Influenza A peaked during weeks 51 and 52 of 2023, consistent with our findings and slightly later than the previous season. ERVISS data indicated the start of the epidemic in week 50 of 2023, with a decline beginning in week 4 of 2024 [23]. In contrast, influenza B peaked in week 8 of 2024 according to the CDC, while our observations noted most cases in week 10, matching ERVISS observations of an earlier decline compared to previous seasons [24].

The 1918 influenza pandemic, the deadliest in modern history, caused 50 million deaths—about eight times the current toll of COVID-19. A similar pandemic could occur again without proper preparedness, as factors like population growth, climate change, and human–animal interactions drive rapid viral outbreaks. Influenza viruses, which are zoonotic and mutate quickly, often create new strains in humans. However, the public often underestimates the seriousness of influenza, confusing it with the common cold due to its seasonal nature. This underestimation leads experts to believe that a major influenza pandemic is almost inevitable [25]. Therefore, maintaining a comprehensive influenza surveillance system is crucial. Annual vaccinations, updated based on surveillance data, are recommended to ensure that public health responses remain effective and adaptable to the evolving nature of influenza viruses [26].

Additionally, during the winter season of 2023–2024, we identified a limited number of HAdV cases, consistent with previous seasons in which low levels of adenovirus were observed without clear seasonality [27]. It is important to note that HAdV surveillance is limited, as it is not a notifiable disease [26]. Infections with HAdV present with symptoms similar to other flu-like illnesses, such as influenza and SARS-CoV-2, and there is no specific treatment available [28]. Consequently, clinicians do not routinely order HAdV testing, and individuals with mild symptoms often do not seek medical care [28]. These factors contribute to the underreporting and limited understanding of the true incidence and seasonality of HAdV infections [28]. Our findings highlight the need for more comprehensive surveillance and reporting to better understand the epidemiology of HAdV.

To continue, on 5 May 2023, WHO declared an end to the COVID-19 pandemic [29]. However, the seasonality of COVID-19 is still under study and debate. While there is some evidence of seasonal patterns, they are not as distinct as those seen in viruses like influenza or RSV. During the first years of the pandemic, multiple infection waves were more influenced by new variants (e.g., Delta and Omicron) and changes in public health measures rather than clear seasonal trends. Factors such as limited years of SARS-CoV-2 circulation, pandemic dynamics, environmental conditions, human behavior, public health measures, and vaccination campaigns have all affected the virus’s spread, potentially obscuring any underlying seasonality [30].

Our results suggest that as COVID-19 becomes endemic, there may be a seasonal pattern to SARS-CoV-2 infections, with a higher incidence in the late fall and winter months. These results align with other studies showing seasonal spikes in COVID-19 cases, hospitalizations, and mortality from November through April, consistent with typical winter respiratory virus patterns [18]. However, it is unclear if enough time has likely elapsed for SARS-CoV-2 to completely transition to stable endemic seasonality. Ongoing research is needed to fully understand the potential seasonality of COVID-19, which will help develop more effective and targeted public health policies, such as well-timed booster vaccination drives, COVID-19 screening initiations, and nonpharmacological interventions [30].

It is necessary to highlight though that if SARS-CoV-2 becomes an endemic coronavirus and exhibits similar seasonality patterns to influenza and RSV, this would lead to overlapping seasons in temperate regions and put considerable additional strain on healthcare systems during the winter season [31].

Despite being a significant respiratory pathogen in infants, RSV is often overlooked in seniors and vulnerable adults. Nearly two-thirds of hospitalized patients are not tested for RSV, including even at-risk groups like the immunocompromised or those with COPD [32]. Physicians often exclude RSV from the differential diagnosis of influenza-like illness, partly due to the lack of specific antiviral treatments [32]. Additionally, the detection of RSV infections is further complicated by the lack of a uniform clinical case definition and the non-specific nature of RSV symptoms [33]. Systematic testing for RSV is crucial to fill gaps in its epidemiology, accurately estimate its true burden, and inform vaccination policies and healthcare preparedness.

The seasonality of respiratory syncytial virus (RSV) infection is also an area of limited knowledge, and the exact global burden of RSV infections remains unknown. RSV seasonality varies depending on both geographical location and climatic conditions. A large study covering 12 seasons and 2030 RSV patients, showed that the season lasted from December to April, with the highest incidence between January and March [34]. Post-COVID-19, RSV infections may surge due to decreased population immunity, particularly affecting adults aged 60 and older, increasing healthcare utilization and the economic burden [35]. The COVID-19 pandemic disrupted the typical seasonal pattern of RSV in the U.S. Previously, RSV seasons (2017–2020) ran from October to April, peaking in December. However, the 2020–2021 season saw no winter epidemic, with a delayed season starting in May 2021, peaking in July, and ending in January 2022. The 2022–2023 season began in June and peaked in November, indicating a return to usual seasonality [36]. For the 2023–2024 season, ERVISS data showed RSV activity rising around week 41 and peaking in week 50, 2023, while our results indicated a peak in late February (week 8, 2024) [23].

Monitoring RSV seasonality is important for timing immunoprophylaxis and evaluating new immunization products. It will be interesting to observe the seasonal patterns of RSV after the administration of the RSV vaccine, which became available in May 2023, as most of the population was not vaccinated for the 2023–2024 season [23].

Diagnosing infectious respiratory diseases in Emergency Departments (EDs) and senior care facilities has been challenging due to ambiguous symptoms and the difficulty in differentiating viral from bacterial infections. Implementing rapid tests in EDs is crucial as they provide immediate results, allowing for prompt and appropriate treatment. This reduces antibiotic overuse, improves patient outcomes, and optimizes resource utilization, ensuring that critically ill patients receive timely care. Rapid tests are also cost-effective, lowering financial burdens on healthcare facilities and patients. Additionally, they help to quickly identify infections, facilitating necessary isolation protocols and reducing the risk of hospital-acquired infections [37]. Simultaneous diagnosis of multiple pathogens using specific rapid tests is crucial, as it allows the immediate identification and differentiation of the main respiratory infections. Rapid antigen tests offer a valuable alternative for quick diagnosis, particularly in healthcare facilities beyond hospitals where molecular testing might not be available.

To conclude, infection control surveillance systems are often underutilized in Greece. A recent study highlighted that the Greek healthcare system struggles to establish proper surveillance for tracking infections and antibiotic resistance [38]. This inefficiency is particularly problematic when it comes to respiratory infections, where timely and accurate data collection can make all the difference in controlling outbreaks. The Greek Infection Prevention Program (GRIPP-SNF), running from 2021 to 2026, aims to transform 10 of Greece’s largest public hospitals into models of best practice for infection prevention and control (IPC) by improving surveillance systems.

A limitation of the present study could be the fact that individuals presenting at hospital EDs with respiratory infection symptoms are supposed to have more severe disease than those choosing to stay home or those visiting primary healthcare (PFC) facilities, setting a bias. However, the truth may differ, as PHC facilities in Greece are often undervalued. Greece’s public healthcare includes rural and urban health centers and private clinics intended as the first point of contact for non-emergency medical care [39]. Several factors contribute to patients bypassing PHC facilities, including lack of access, unfamiliarity with services, and a public perception that hospitals provide better care. Given that Greece has a shortage of hospital beds and healthcare providers compared to other European countries, this can lead to overcrowding and delays in care, particularly during periods of high demand [40]. By strengthening primary care services and reassuring the public, healthcare systems can better manage patient flow, reduce unnecessary hospital burdens, and ensure that individuals receive timely and appropriate care for their health needs.

## Data Availability

The data that support the findings of this study are available on request from the corresponding author.
